# Modification of Surface and Subsurface Properties of AA1050 Alloy by Shot Peening

**DOI:** 10.3390/ma14216575

**Published:** 2021-11-01

**Authors:** Yasemin Yıldıran Avcu, Berkay Gönül, Okan Yetik, Fikret Sönmez, Abdulkadir Cengiz, Mert Guney, Egemen Avcu

**Affiliations:** 1 Department of Mechanical Engineering, Kocaeli University, Kocaeli 41001, Turkey; yaseminyildiran89@gmail.com (Y.Y.A.); berkaygonul@gmail.com (B.G.); oknyetik@gmail.com (O.Y.); avcuegemen@gmail.com (E.A.); 2 Department of Mechanical Engineering, Hasan Ferdi Turgutlu Faculty of Technology, Manisa Celal Bayar University, Manisa 45140, Turkey; sonmezfikret@gmail.com; 3 Department of Automotive Engineering, Kocaeli University, Kocaeli 41001, Turkey; akcngz@gmail.com; 4 Department of Civil and Environmental Engineering, The Environment and Resource Efficiency Cluster (EREC), Nazarbayev University, Nur-Sultan 010000, Kazakhstan; 5 Ford Otosan Ihsaniye Automotive Vocational School, Kocaeli University, Kocaeli 41650, Turkey; 6 School of Materials, The University of Manchester, Manchester M13 9PL, UK

**Keywords:** crack growth, mechanical behaviour, plastic deformation, shot peening, soft metals

## Abstract

AA1050 Al alloy samples were shot-peened using stainless-steel shots at shot peening (SP) pressures of 0.1 and 0.5 MPa and surface cover rates of 100% and 1000% using a custom-designed SP system. The hardness of shot-peened samples was around twice that of unpeened samples. Hardness increased with peening pressure, whereas the higher cover rate did not lead to hardness improvement. Micro-crack formation and embedment of shots occurred by SP, while average surface roughness increased up to 9 µm at the higher peening pressure and cover rate, indicating surface deterioration. The areal coverage of the embedded shots ranged from 1% to 5% depending on the peening parameters, and the number and the mean size of the embedded shots increased at the higher SP pressure and cover rate. As evidenced and discussed through the surface and cross-sectional SEM images, the main deformation mechanisms during SP were schematically described as crater formation, folding, micro-crack formation, and material removal. Overall, shot-peened samples demonstrated improved mechanical properties, whereas sample surface integrity only deteriorated notably during SP at the higher pressure, suggesting that selecting optimal peening parameters is key to the safe use of SP. The implemented methodology can be used to modify similar soft alloys within confined compromises in surface features.

## 1. Introduction

Most pure metals exhibit corrosion resistance [1,2,3], high specific strength [2,4], and good electrical conductivity [2,5] while showing poor mechanical properties [6,7] compared to their alloys. The industrial use of pure metals is strongly dependent on their mechanical properties such as σ_y_, elastic modulus, and hardness [8]. However, most commercial pure metals have relatively poor σ_y_ [2,9], σ_u_ [10], surface hardness [8], and wear resistance [8], which may restrict their usage, particularly in applications requiring moderate mechanical performance [2,4,6,11]. Specifically, their surface hardness could be very low as available strengthening mechanisms within their microstructure are limited due to a lack of alloying elements [4,6,7,12,13], which may restrict their widespread use in surface-related applications.

Commercially pure Al, including AA1050 Al alloy, has been widely used in various applications such as household items [11], food containers [3], chemical plant equipment [3], light reflectors [3,14], rivets [15], heat exchangers [14], and electrical wiring applications [16]. They exhibit high corrosion resistance [17,18] and high thermal and electrical conductivity [2,5,18]. The mechanical properties of commercially pure Al alloy (e.g., AA1050-H14 [3]) can be listed as follows: σ_u_ of 78–107 MPa [8,14,19], σ_y_ of 74–128 MPa [8,19], elastic modulus of 68–71 GPa [3,19], and hardness of 25–30 HV [8,20]. These mechanical properties are relatively poor compared to their alloyed counterparts (e.g., AA2020-T4 [21], AA2024 [22], 5056 Al alloy [23], AA7075 [24,25]) and thus restrict their usage in applications with moderate stress, indentation, and friction [10,14,18]. Consequently, there has been a growing interest in improving the mechanical properties (particularly hardness) of pure Al alloy with an acceptable compromise in other physicochemical properties [2,26] by using different strategies such as SPD methods [5,17,20,26] and incorporation of nanoparticles into pure Al [8,10]. Besides, improving surface and sub-surface hardness of 1xxx Al alloys (AA1050, AA1070, AA1100) is particularly important since their marginal tribological properties associated with their low hardness remarkably restrict their usage in wear-related applications such as architectural flashings, cooking utensils, and rivets [27,28,29,30].

Some SPD methods, including ARB [17], ECAP [20], and high-pressure torsion [31,32], were successfully used to enhance the mechanical properties of pure Al. Furthermore, AMCs reinforced with carbonaceous nanoparticles such as graphene and carbon nanotubes exhibit improved mechanical properties [10]. However, SPD methods are primarily bulk deformation processes, which usually require expensive machinery [33] and great forces [31,34] and have limitations on geometry [6,26,31,33]. Besides, the research on processing AMCs has been limited by the challenges related to AMC production. For instance, it is still challenging to homogeneously distribute the reinforcing material within the pure Al microstructure, limiting the improvement of mechanical properties and causing non-uniform microstructure [10]. There is a need to develop robust approaches to improve the mechanical properties of Al with ease and consistency, which will widen its use.

SP is a well-known mechanical surface treatment method to improve the fatigue life of materials used in various engineering applications (e.g., automotive, aerospace, and biomedical applications) [1,24,25,35,36,37,38,39,40]. More specifically, it is an enticing process for enhancing mechanical properties (i.e., hardness, roughness, residual stress, σ_y_ [1,6,22,23,41,42,43]), corrosion resistance [34,35,42,44,45], electrical conductivity [14], wear resistance [24], and biological properties [6,46,47] of engineering alloys. Briefly, SP modifies the surface and subsurface features of materials through plastic deformation caused by the bombardment of small steel shots onto a surface [22,24,40,42,46,47,48,49], improving the fatigue life by inhibiting micro-crack formation and propagation [1,23,36,37,38,39,50,51,52]. The literature on improving the mechanical behaviour of pure metals using SP is somewhat limited [1,6,7,9,41,47,53,54]. Zhu et al. [53] showed enhanced surface hardness for shot-peened pure titanium at different peening parameters (shot size, distance, and duration). Dai et al. [9] showed that increasing SP duration resulted in increased hardness and decreased elongation of pure titanium, attributed to the work hardening and formation of nanocrystals on the surface. SP of pure titanium at different Almen intensities resulted in obtaining a fine-grained microstructure [54]. Similar results were also reported for other pure metals. For instance, Li et al. [41] reported that the hardness of pure Cu can be increased down to 400 µm in-depth with SP. Other studies showed that SP could improve the mechanical properties of pure copper [6,7,41] and pure titanium [1,9,47,53,54].

Studies on improving the mechanical properties of industrially pure Al alloy AA1050 by SP are limited. Cho et al. [22] studied the surface hardening mechanism of shot-peened AA2024 Al alloy, presenting a hardness increase associated with SPD for Al alloys. Gariepy et al. [38] demonstrated microstructural modifications in shot-peened AA2024 alloy using electron backscatter diffraction, showing high dislocation densities within the SP affected regions down to ~70 μm. Studies showing the beneficial influence of SP on the corrosion resistance of Al alloy are also available [42,44,45]. Considering the summarised literature on SP of pure metals, improved mechanical properties (more specifically fatigue behaviour, tensile strength, and yield strength) and tribological properties (e.g., abrasion resistance) can be achieved by modifying the surface and sub-surface features (particularly hardness) of AA1050 alloys (as well as other 1xxx Al alloys) via SP. However, studies are limited on the utilisation of SP to modify the properties of pure Al alloys. Thus, the present study aims to report on the variation in both hardness and surface/subsurface features (including microstructure, roughness, and material removal) of shot-peened commercial pure AA1050 Al alloy under different peening pressures and cover rates.

## 2. Materials and Methods

### 2.1. Materials

Commercial AA1050 Al alloy sheets (thickness: 6 mm) were supplied from Assan Aluminium Industry and Trade Inc. (Kocaeli, Turkey) (chemical composition and mechanical properties provided in Table 1). The sheet metal was cut into the smaller samples with dimensions of 76 mm × 25 mm × 6 mm. Afterward, homogenisation annealing was carried out at 400 °C for 4 h to improve the microstructural homogeneity of the samples before SP. Finally, the samples were ground and polished, and the initial surface roughness and hardness of the samples were measured (0.3 µm and 27 HV, respectively).

### 2.2. Shot Peening Process

**Figure 1:**
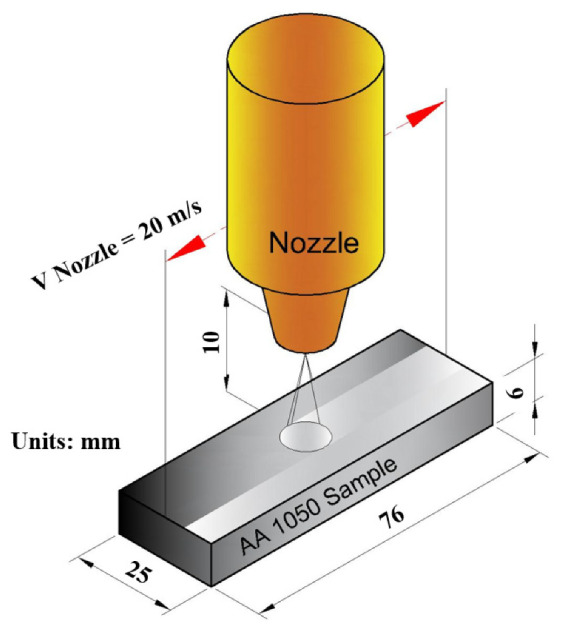
Schematic illustration of the shot-peening of AA1050 alloy samples.

SP was performed on the metallographically prepared AA1050 Al alloy samples with stainless-steel shots (diameter: 0.7–1.0 mm (type: Chronital S60), shot hardness: 450 HV) as detailed elsewhere [55,56]. Figure 1 shows the SP of AA1050 by highlighting some of the important parameters and dimensions. It was carried out using a custom-designed automatic-controlled SP system including an air compressor, a dehumidifier, a pressure regulator, and a blasting cabinet along with other related pneumatic equipment such as valves and pipes. The peening parameters were: impingement pressure of 0.1 and 0.5 MPa, surface cover rate 100% and 1000%, impingement angle of 90°, a working distance of 10 mm, and a nozzle speed of 20 mm/s. Finally, the peened samples were ultrasonically cleaned in alcohol for 10 min, and the variation in the mass of the peened samples was measured using an electronic balance (accuracy: ±0.1 mg). A robust image analyzing method was applied to the peened samples using optical microscope images and ImageJ^®^ (version 1.52p, 2021, National Institutes of Health, Bethesda, MD, USA) to determine the peening time needed to reach the full cover rate at peening pressures of 0.1 and 0.5 MPa. Then, the peening time was varied to achieve 100% and 1000% cover rates, where the cover rate was adjusted by estimating the peening time using the time needed for full coverage (100%) [35,57,58]. A minimum number of five samples were prepared and tested.

### 2.3. Subsurface Microstructural and Mechanical Characterisation

The shot-peened samples were cross-sectioned with a diamond cutting disc using a precision cutter, and then the cut specimens were moulded into a resin. Afterward, the moulded specimens were ground (#320, #600, #1200, and #2000 grits) and polished (1 and 3 µm diamond suspension) using an automatic metallographic sample preparation system. The polished specimens were cleaned for 10 min with alcohol in an ultrasonic bath. The cleaned specimens were investigated under an SEM (Tescan Vega 2, Brno-Kohoutovice, Czechia). Finally, microhardness tests were performed (HV_0.05_, 15 s, and five replicates) using a hardness tester (Zeiss micro Vickers hardness tester, Oberkochen, Germany).

### 2.4. Surface Characterisation

The shot-peened samples were cleaned via ultrasonication in alcohol for 10 min. Afterward, the surface roughness of the samples was measured using a surface profilometer (Mitutoyo Surftest SJ-301, Kanagawa, Japan) by using the following parameters: cut-off wavelength (λ_c_) of 0.8 mm, evaluation length (EVA-L) of 4.0 mm, and cut-off filter of Gaussian. The arithmetic average roughness (Ra) of the shot-peened and unpeened specimens were calculated using (1), which gives the average of all peaks and valleys of the roughness profile [59].

(1) $$R_{a}=\frac{1}{lr}\int_{0}^{lr}\left|z\left(x\right)\right|dx$$

Furthermore, the peened surface morphologies were investigated under an SEM equipped with an EDS (Tescan Vega 2, Brno-Kohoutovice, Czechia, with Oxford Instruments EDS detector, High Wycombe, UK). The SEM images of shot-peened surfaces were post-processed to quantify the area covering the embedded shots as a function of SP parameters using ImageJ^®^ (version 1.52p, 2021, National Institutes of Health, Bethesda, MD, USA) [60]. Further, the size distribution of the embedded shots was obtained by analysing the SEM images by following a similar methodology used in our previous study [61]. Briefly, backscattered electron images over a region of 100 μm × 100 μm were randomly taken from the shot-peened samples, and then embedded shots coverage and the distribution of shot size were analysed using image analysis (ImageJ^®^) [62].

## 3. Results

### 3.1. Hardness Improvement after SP

**Figure 2:**
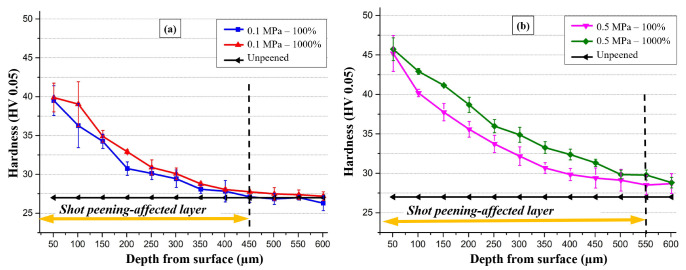
Change in hardness along depth under different cover rates at peening pressures of (**a**) 0.1 MPa and (**b**) 0.5 MPa.

Figure 2 shows the microhardness variation through the depth from the surface of the homogenised and shot-peened AA1050. After SP, hardness improved on the surface and subsurface (48% and 70% for peened samples at 0.1 MPa and 0.5 MPa, respectively) where hardness gradually diminished with respect to the distance from the surface, in parallel to results reported in the literature on SP of AA7075 [24] and 6061 Al [42] alloys. Hardness reached a maximum of 46 HV after SP (0.5 MPa and 1000% cover rate), and it slightly increased with increasing cover rate. The surface hardness increased by 22% at higher pressure, whereas the cover rate had an insignificant effect on the hardness at 0.1 MPa and a slight effect at 0.5 MPa. Thus, it was demonstrated that peening pressure was the key parameter affecting hardness compared to the cover rate (Figure 2).

The SP-affected layer is a measure of the intensity of the plastic deformation that occurred due to the high kinetic energy of the impact of shots [42]. The depth of the peening-affected zone is around 450 µm and 550 µm for peening pressure of 0.1 MPa and 0.5 MPa, respectively (Figure 2). The maximum microhardness observed at the surface decreases rapidly with an increase in depth ~50–100 µm below the surface.

### 3.2. Modification of Surface Morphology and Subsurface Microstructure

**Figure 3:**
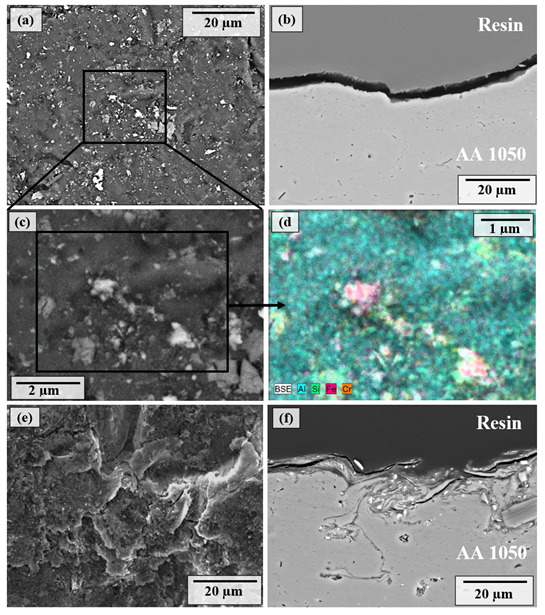
SEM images of (**a**) surface morphologies, (**b**) cross-sectional microstructures, (**c**) high-magnification image at the region of interest, (**d**) EDS analysis of shot-peened AA1050 alloy at 0.1 MPa and 100% cover rate, (**e**) SEM images of surface morphologies, and (**f**) cross-sectional microstructures of shot-peened AA1050 alloy at 0.1 MPa and 1000% cover rate.

**Figure 4:**
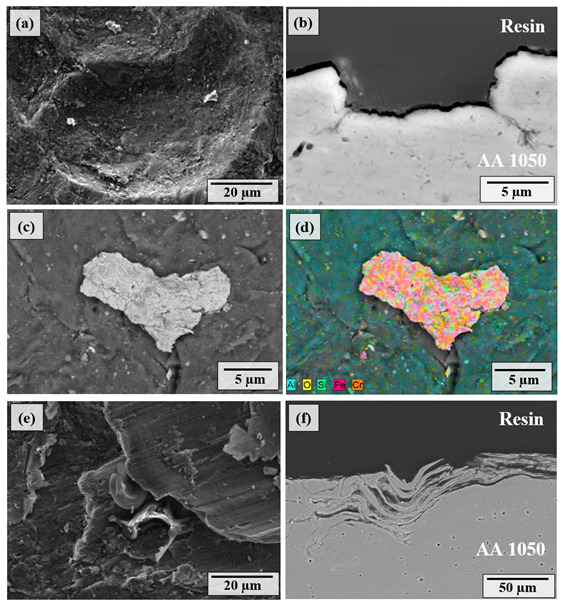
SEM images of (**a**) surface morphologies, (**b**) cross-sectional microstructures, (**c**) high-magnification image at the region of interest, (**d**) EDS analysis of shot-peened AA1050 alloy at 0.5 MPa and 100% cover rate, (**e**) SEM images of surface morphologies, and (**f**) cross-sectional microstructures of shot-peened AA1050 alloy at 0.5 MP and 1000% cover rate.

Surface and cross-sectional SEM images of the shot-peened samples (Figure 3 and Figure 4) revealed the modifications in surface morphology and subsurface microstructure as a function of SP pressure and cover rate. Even at the lower SP pressure (0.1 MPa), the surface of the samples is plastically deformed considerably, exhibiting dents and ridges caused by indentation by shots [35,45,63] (Figure 3a,b). The cross-sectional microstructure was crack-free at 0.1 MPa and 100% cover rate, proving that the surface integrity of the samples was generally maintained. Some residues of shots embedded onto the shot-peened surface are observed by EDS (Figure 3c,d), similar to the observations as reported in the literature [38]. The embedded shots are less than 1 μm in diameter, possibly fragmented and embedded onto the surface due to repeated impact of shots. The plastic deformation becomes more severe and distinct with increasing cover rate up to 1000%, where microcracks are formed beneath the surface down to a depth around 19.5 μm (Figure 3e,f). Furthermore, a significant material pile-up at the surface occurs at a 1000% cover rate (Figure 3e), which can be attributed to the SPD of the surface. Thus, SP at a 1000% cover rate deteriorates the surface due to repeated impact of shots (Figure 3e,f).

The surface and cross-sectional SEM images of the shot-peened samples at 0.5 MPa and cover rates of 100% and 1000%, along with the corresponding EDS analysis (Figure 4), show that even at 100% cover rate, large craters were formed on the surface due to the high impact energy of the shots at 0.5 MPa (Figure 4a), associated with SPD. The cross-sectional image (Figure 4b) illustrates the damage beneath a crater formed during SP (0.5 MPa, 100% cover rate). Despite the compressive residual stress formed by SP (which could restrict and retard crack formation and propagation), some micro-cracks formed just beneath the formed crater (Figure 4b). The measured size of the microcracks was around 3–4 µm in length and 0.2–0.4 µm in width, indicative of the SPD that occurred by SP at 0.5 MPa.

A large fragment of shots embedded onto the surface can be seen in Figure 4c, also confirmed by the EDS analysis (Figure 4d). This proves that the penetration of the shots is higher at 0.5 MPa compared to that of 0.1 MPa as larger shot fragments can be embedded onto the surface. The material pile-up is more evident in the cross-section of shot-peened AA1050 at 1000% (Figure 4f), as previously described by so-called folding mechanisms in the literature [64]. Thus, the severity of the plastic deformation remarkably increases with increasing cover rate. Eventually, micro-voids formed down to a depth of around 50 µm, clearly showing that the alloy loses its surface integrity which may detrimentally affect the mechanical properties of the surface. Besides, significant material removal may occur if the number of micro-voids further increases. The formation of micro-voids is due to folding mechanisms associated with SPD that occur at high SP pressure and cover rate.

The modified surface morphology and subsurface microstructure depending on SP pressure and cover rate (Figure 3 and Figure 4) are mostly in agreement with the variation in hardness as a function of SP pressure and cover rate (Figure 2). More particularly, the increase in plastic deformation at the higher pressure agrees well with the increase in hardness at 0.5 MPa. However, SPD at 1000% cover rate at both 0.1 and 0.5 MPa does not yield a notable increase in hardness while leading to the formation of micro-cracks and micro-voids (Figure 3 and Figure 4), showing the deterioration of the surface at higher cover rates. Thus, overall, optimising the SP pressure is the key to enhancing the hardness, whereas increasing the cover rate is ineffective to increase the hardness while detrimentally affecting the surface integrity.

**Figure 5:**
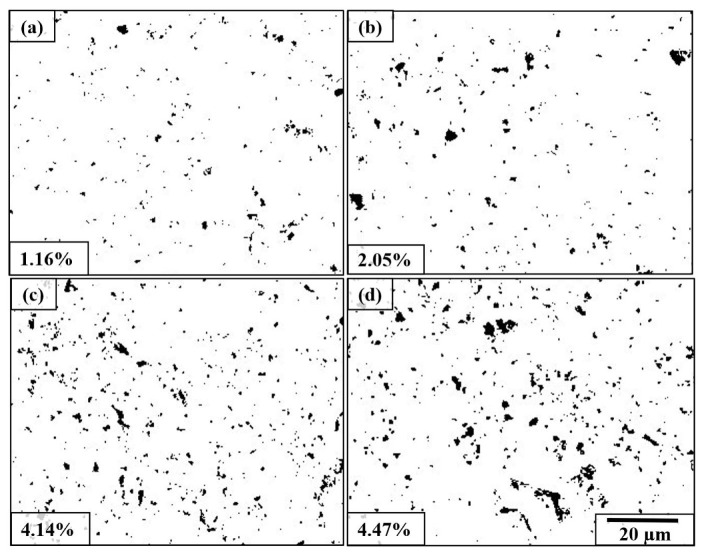
Areal coverage rate of embedded particles due to SP at (**a**) 0.1 MPa and 100% cover rate, (**b**) 0.1 MPa and 1000% cover rate, (**c**) 0.5 MPa and 100% cover rate, and (**d**) 0.5 MPa and 1000% cover rate. The black regions are the embedded shots, and the white background is the AA1050 alloy.

**Figure 6:**
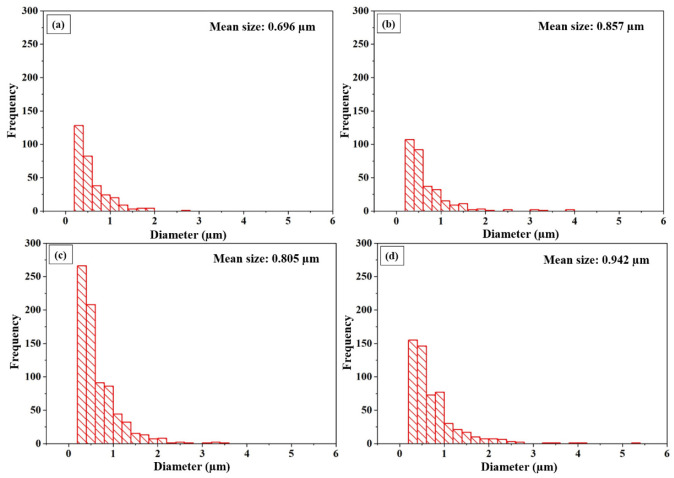
Size distribution histograms of embedded shots onto surfaces of shot-peened AA1050 alloy at (**a**) 0.1 MPa and 100% cover rate, (**b**) 0.1 MPa and 1000% cover rate, (**c**) 0.5 MPa and 100% cover rate, and (**d**) 0.5 MPa and 1000% cover rate.

Figure 5 reveals the areal coverage of embedded shots onto the AA1050 surface shot-peened at different parameters, where black regions are embedded stainless-steel shots, and the white background is the AA1050. Figure 6 shows the distribution and the mean size of embedded shots. The embedment was almost double at 1000% compared to 100% at 0.1 MPa shot pressure (Figure 5a,b). Furthermore, the number and the mean size of the embedded particles increased at the higher cover rate (Figure 6a,b). The areal coverage rate of the embedded particles also increased at the elevated pressure (0.1 MPa vs. 0.5 MPa), agreeing well with the corresponding surface morphologies (Figure 3 and Figure 4). The number and the diameter of the embedded shots increased at 0.5 MPa (from 0.696 μm to 0.942 μm in diameter), probably due to the higher impact energy of the shots at that pressure.

**Figure 7:**
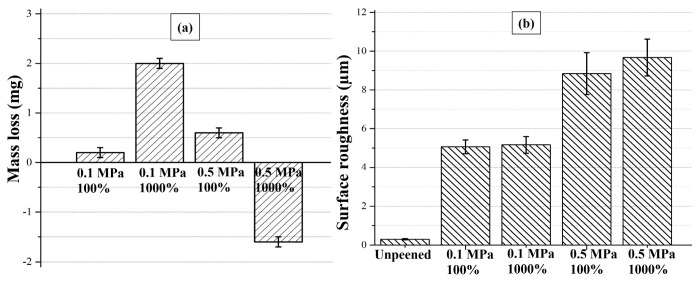
(**a**) Mass loss and (**b**) arithmetic average surface roughness (Ra) as a function of SP pressure and cover rate.

To further understand the variation in surface integrity as a function of SP pressure and cover rate, the variation in mass loss and Ra was illustrated (Figure 7). At 0.1 MPa, mass loss increases with the higher cover rate, most probably due to the removal of plastically deformed surface features (e.g., ridges of formed craters) by the repeated impact of shots. By contrast, mass loss decreases with the higher cover rate at 0.5 MPa, indicating that the embedding of shots is more predominant at 0.5 MPa due to the higher impact energy of shots. The variation in mass loss agrees well with previously discussed surface morphologies as a function of SP pressure and cover rate (Figure 3 and Figure 4). The Ra of shot-peened surfaces is remarkably higher than that of the unpeened surface (Figure 7b), clearly proving the surface modification with SP. The Ra is around 5 µm at 0.1 MPa (while around 9 µm at 0.5 MPa), agreeing well with the corresponding SEM images of surface morphologies and cross-sectional microstructures. Considering the standard deviation of Ra shown (Figure 7b), the influence of the cover rate on Ra can be ignored.

## 4. Discussion

### 4.1. Mechanical Properties of Shot-Peened AA1050 Alloy

Mechanical properties of pure metals (i.e., pure Al alloys such as AA1050 and AA1070 alloys) are mostly to be improved through grain refinement (i.e., Hall–Petch strengthening) since other strengthening mechanisms (i.e., solution hardening, precipitation hardening) cannot be exploited due to their chemical composition [65]. Such grain refinement increases dislocation density within microstructure by inhibiting the dislocation mobility during plastic deformation [66], leading to strain hardening [67]. Thus, enhancing the mechanical properties of pure Al alloys (e.g., AA1050, AA1070, and AA1100) through the aforementioned strengthening mechanisms using SPD methods is gaining increasing attention in recent years [65,66,67,68,69]. Other mechanical properties (e.g., *σ_u_*, *σ_y_*) may not be fully correlated with hardness as it is not an intrinsic material property that depends on the measurement method and various other material features. However, it is still possible to relate the changes in the hardness of a material with its other mechanical properties [70,71]. For instance, a relationship between $H_{V}$, *σ_y_*, and σ_u_ was proposed for AA7050 alloy [70]. Several assumptions for the prediction of strength–hardness for polycrystalline materials (e.g., (2, 3)) were also suggested that define the relationship between $H_{V}$, $\sigma_{Y},$ and $\sigma_{U}$ [71].

(2) $$\frac{\sigma_{Y}}{H_{V}}=3$$

(3) $$\frac{\sigma_{U}}{H_{V}}=3.45$$

Changes in hardness of shot-peened AA1050 alloy under different peening parameters were examined as a function of depth (Figure 2) to discuss the mechanical improvement of the alloy with shot-peening. The mechanical properties of the surface and subsurface were significantly improved by SP (Figure 2) due to the aforementioned strengthening mechanisms. Briefly, SP improves the hardness of metals by refining the grain size and increasing the dislocation density within the peening-affected zone through localised SPD [54], activating the aforementioned strengthening mechanisms (i.e., Hall–Petch strengthening) [63]. Furthermore, forming a nanocrystalline layer with high dislocation density beneath the shot-peened surface improves microhardness [58]. This improvement is more pronounced at higher peening pressures (Figure 2b) since the deformation energy of the shots that can be transferred to the material is directly related to their kinetic energy (i.e., SP pressure) [24,42,52,53,56,58,64,72]. The increasing coverage rate of the embedded shots and the size of the shots supports that the impact energy of the shots is higher at the elevated pressure (Figure 5 and Figure 6). However, the effect of cover rate on the near-surface hardness (50 μm beneath the surface) is almost insignificant, resulting in a hardness increase of less than %2 for both pressures (Figure 2). This is probably due to the saturation of hardness in the near-surface at a 100% cover rate for both pressures since hardening is a consequence of plastic deformation [52]. The hardening is commonly linked to the grain refinement and dislocation entanglement for AA1050 subjected to SPD [20], as discussed earlier. Thus, hardness saturation may occur if the ultimate grain size and dislocation density are reached within the near-surface for those parameters, where the plastic deformation caused by the impact energy of the shots is mostly related to the peening pressure rather than the cover rate, as discussed previously [52]. Therefore, increasing the cover rate (i.e., processing time) cannot further increase the hardness of the region very close to the surface due to the saturation of hardness (Figure 2). Similar results were also reported in the literature on examining the effect of processing time (i.e., cover rate, cycle, and pass number) on the hardness of severe plastic deformed materials by SP and other SPD methods, where the hardness increase in near-surface is either very limited or insignificant with increasing processing time due to the saturation of hardness [52,73].

The hardness of the shot-peened samples at 0.5 MPa was almost double that of the unpeened samples. Besides, the increase in surface roughness (Figure 7b) and changes in the surface morphology (Figure 3 and Figure 4) at the elevated peening pressure are indicative of the larger plastic deformation of the alloy at the higher peening pressure, leading to improved hardness. The improvement in hardness gradually decreases with increasing depth (Figure 2), in parallel to the literature on SP of 2024 Al alloy [74], 6061 Al alloy [42], Ti6Al4V alloy [56], ferrite–pearlite steels [63], pure copper [6], and AZ31 magnesium alloy [4]. The influence of the cover rate on hardness is not notable at the lower peening pressure, where it slightly enhances the hardness at high peening pressure (Figure 2b). Wu et al. [75] similarly observed that SP on 18CrNiMo7–6 steel slightly increased surface hardness (up to 7.2%) with increasing cover rate. Finally, Wang et al. [63] showed that an increased cover rate (from 100% to 18.000%) could impart more severe and more profound plastic deformation to the surface layer, increasing the microhardness and thickness of the SP-affected layer in pearlite steel.

**Figure 8:**
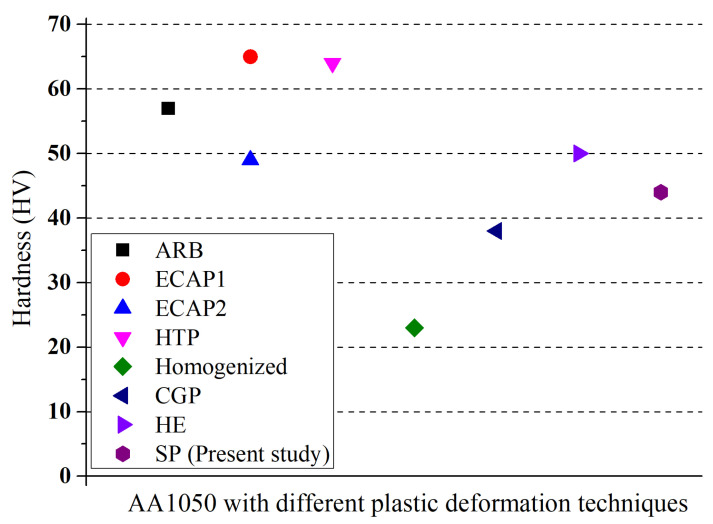
Comparison of obtained hardness values of AA1050 through ECAP1 [20], ECAP2 [77], HPT [31], ARB [17], CGP [71], HE [77], SP, homogenisation annealing.

Several other SPD methods (e.g., ECAP [20], HPT [31], and ARB [17]) were applied to pure Al (i.e., AA1050) to enhance its mechanical properties, mainly focusing on forming fine-grained microstructural features within the bulk material. Figure 8 compares the obtained hardness values of the shot-peened AA1050 alloy in the present study with those obtained using the widely used SPD methods in the literature. Considering the certain limitations of SPD methods (e.g., challenges related to processing special size and shapes [17,20], applicability on different alloys [20], and obtaining a homogeneous microstructure [31]), SP seems to be a promising method to enhance the surface and near-surface hardness of AA1050 alloy even though it provides relatively lower hardness improvement compared to other methods. Further, SP has numerous advantages over those SPD methods, including simple equipment requirements, adjustable surface and subsurface properties by variation in process parameters, low energy consumption, short process time, application of complex shapes of the workpieces, and high efficiency production efficiency [4,52,76].

Pure Al and its alloys have replaced engineering conductor materials (e.g., copper and its alloys) in various applications (e.g., transmission lines, conductors, and power cables) due to their lightweight and high electrical conductivity [78,79]. The commercially pure Al alloys (i.e., AA1050, 1070, 1100) also have notably higher electrical conductivity compared to other Al alloys (e.g., 60.3% IACS for AA1050 vs. 26.1% IACS for AA5483 Al alloy [77]). The strengthening mechanisms obtained by increasing the number of defects in a pure Al lattice (i.e., precipitates and solute atoms) significantly reduce the electrical conductivity [80]. Therefore, it is crucial to enhance the mechanical properties of 1xxx Al alloys considering the demand for electrically conductive Al alloys with better mechanical properties [78]. In summary, the proposed SP approach to enhance the mechanical properties of the AA1050 alloy seems promising to widen its practical application, which is currently limited mainly due to its poor mechanical properties [78].

### 4.2. Surface and Subsurface Features of Shot-Peened AA1050 Alloy

**Figure 9:**
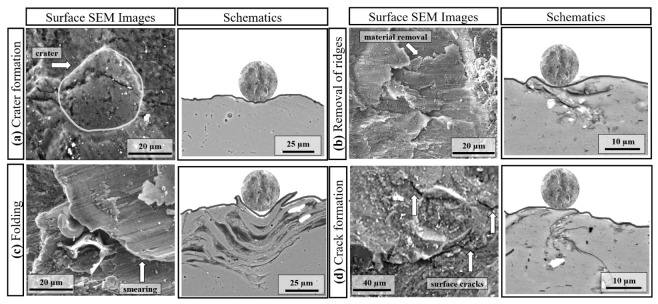
Schematics of deformation mechanisms; (**a**–**d**) showing the interaction of the shots with the surface under different parameters, as observed by SEM analysis.

As underlined in the *Introduction*, most of the studies related to the shot-peening of engineering alloys (i.e., Al alloys, titanium alloys, copper alloys) only discuss their mechanical properties (particularly fatigue behaviour and residual stress) and microstructural features such as grain size and orientation. In contrast, studies revealing changes in surface and sub-surface features are limited. For instance, the embedment of shots onto the shot-peened materials has not been thoroughly analysed in the literature. Here, the changes in the surface features (e.g., surface morphologies, roughness, mass loss, area coverage, and size distribution of embedding particles) as a function of SP pressure and cover rate were analysed and discussed in detail. Figure 9 illustrates the deformation mechanisms that occur during the SP process under different parameters along with the surface and cross-sectional microstructures.

Surface craters (i.e., dimples, indents) were formed due to SPD induced by the repeated impact of the shots (Figure 9a) [63]. The craters are nearly circular, and their size and depth vary on peening pressure. The surface roughness significantly increased at the higher pressure since the crater formation resulted in an uneven surface (Figure 7b) [40]. Crater formation was the active deformation mechanism that modified the surface and changed the roughness via the formation of irregular valleys and peaks in the surface profile, similar to the results reported in the literature [4,25,55,56,63]. The influence of cover rate on the surface roughness was insignificant at the low pressure, whereas the surface morphology significantly changed at the higher cover rate and pressure. The repeated impacts of shots at high cover rates may: (1) break the edges of the formed craters [63] (Figure 9b), (2) cause peeling and folding of the surface [64] (Figure 9c), and (3) form surface and subsurface cracks (Figure 9d). The first mechanism may reduce roughness, whereas the second and third may increase it. For soft metals, the second mechanism is expected to be the dominant one [64], which could explain the small increase in roughness observed at the higher cover rate, particularly at the higher peening pressure (Figure 7b). In order to infer which mechanism is dominant in modifying the surface morphology and roughness during SP, areal roughness measurements and further morphological analyses are needed to understand better the changes in surface roughness and texture as a function of peening parameters [59,81].

The mass loss of the shot-peened samples was minimal (Figure 7a), and signs of thin scratches were observed on the peened surfaces (Figure 4e), indicating little wear due to SP. The edges of the craters were broken down via the repeated impact of shots at the higher cover rates, causing the material removal (Figure 9b). The sharp features and surface overlaps (folded surface layers) became more distinct at the higher peening pressure and cover rate, which can form micro-voids and act as crack initiators (Figure 4e,f), indicating an over-peened surface [25]. Finally, a limited embedment of shots is qualitatively and quantitatively shown (Figure 5 and Figure 6). This seems due to the lower hardness of the AA1050 surface compared to that of stainless-steel shots (27 HV vs. 450 HV). The embedment of particles could occur during blasting (i.e., SP [60] and solid particle erosion [82]), where the intensity of the embedment depends on particle size, impact energy, and mechanical properties of the shots and the targeted material [83]. The areal coverage of embedded shots increases at the higher peening pressure since the impact energy of the shots is linked to the peening pressure (Figure 5). The increasing cover rate only increases the number of particles that collide with the surface, significantly increasing the areal coverage of embedded shots at 0.1 MPa (Figure 5a,b). However, the effect of cover rate on the areal coverage of embedded shot is not very significant at 0.5 MPa as less than a 10% increase occurs with increasing cover rate. This may be due to the intense surface modification (i.e., surface topography and morphology) at 0.5 MPa (Figure 4 and Figure 7), which may inhibit the embedding of shots due to the deterioration in the surface integrity. However, the cover rate effect is still unclear, while the existing literature on the variation in shot embedment is limited and controversial [84].

## 5. Conclusions

AA1050 Al alloy samples were shot-peened using a custom-designed automatic shot peening (SP) system under different parameters. The modification of surface hardness, roughness, morphology, and cross-sectional microstructure as a function of SP pressure and the cover rate was examined in detail. Additionally, the embedment of shots as a function of SP parameters was qualitatively and quantitatively investigated. Finally, the deformation mechanisms that occur during SP were schematically illustrated and discussed through the surface and cross-sectional scanning electron microscope images.

SP considerably modified the hardness and surface features (i.e., morphology and roughness). The hardness of shot-peened samples was around twice that of unpeened samples. However, micro-cracks and micro-voids became evident along with increased roughness at the higher SP pressure and cover rate, suggesting that optimizing the SP parameters is the key to enhancing the hardness with less compromise in surface integrity. Furthermore, the depth of the hardened surface layer ranged between 200 and 500 μm depending on SP pressure and cover rate, whereas the surface deterioration was limited down to a depth around 50 μm even at the higher pressure (0.5 MPa) and cover rate (1000%). Thus, further mechanical grinding or polishing can be suggested to remove the deteriorated surface layer if the surface deterioration due to SP is inevitable. Limited shot embedment occurred with an areal coverage of embedding shots ranging between 1 and 5%. The number and the mean size of the embedded shots increased with increasing SP pressure. Surface craters (i.e., dimples, indents), folding of the surface, surface and subsurface cracks, and limited material removal occurred on the shot-peened surfaces, which modified the surface morphology and roughness as a function of SP parameters.

The proposed SP methodology can be used to enhance the mechanical properties (i.e., hardness) of soft metals, which may broaden their use to various engineering applications that require moderate mechanical performance (e.g., electrical transmission cables for 1xxx Al alloys). Other mechanical properties (particularly fatigue behaviour) can also be improved with the proposed methodology, resulting from compressive residual stress created by the repeated impact of the shots onto the surface. Furthermore, improving the surface and subsurface hardness by SP without changing the microstructural and mechanical properties of the bulk material may be advantageous compared to severe plastic deformation methods (e.g., equal channel angular pressing, high-pressure torsion) where bulk properties are ultimately affected during processing. Thus, the proposed approach of SP can pave the way to produce surface tailored materials (i.e., soft metals) with enhanced mechanical properties (e.g., hardness, yield strength, tensile strength) and wear resistance for surface-related uses such as architectural flashings, cooking utensils, and rivets, where the use of commercially pure Al alloys is restricted due to their marginal wear resistance associated by their low surface hardness.

## Figures and Tables

**Table 1:** Chemical composition and mechanical properties of AA1050.

Chemical Composition of AA1050 (wt.%)							
Si	Fe	Cu	Mn	Mg	Ti	Zn	Al
0.123	0.259	0.001	0.004	0.003	0.010	0.008	Bal.
Mechanical Properties of AA1050							
Density (kg/m^3^)		Melting Temperature (°C)		Young’s Modulus (GPa)		σ_u_ (MPa)	
2.71		650		71		78	

## Abbreviations

Al	Aluminium
AMC	Al matrix composites
ARB	Accumulative roll bonding
CGP	Constrained groove pressing
ECAP	Equal channel angular pressing
EDS	Energy-dispersive X-ray spectroscopy
HE	Hydrostatic extrusion
HPT	High pressure and torsion
IACS	International annealed copper standard
SEM	Scanning electron microscopy
SP	Shot peening
SPD	Severe plastic deformation

## Symbols

σ_u_	Ultimate tensile strength
σ_y_	Yield strength
H_v_	Vickers hardness
R_a_	Average surface roughness
